# Invasive Aspergillosis in a Patient with Stage III (or 3a or 3b) Non-Small-Cell Lung Cancer Treated with Durvalumab

**DOI:** 10.1155/2019/2178925

**Published:** 2019-08-27

**Authors:** Ashish Gupta, Aung Tun, Katy Ticona, Aam Baqui, Elizabeth Guevara

**Affiliations:** ^1^Internal Medicine, The Brooklyn Hospital Center, Brooklyn, New York, NY, USA; ^2^Hematology Oncology, The Brooklyn Hospital Center, Brooklyn, New York, NY, USA; ^3^Pathology, The Brooklyn Hospital Center, Brooklyn, New York, NY, USA

## Abstract

Durvalumab is a therapeutic monoclonal antibody that blocks the checkpoint inhibitor, programmed death ligand 1 (PD-L1), resulting in T-cell activation and an antitumor response. Durvalumab is approved for patients with unresectable stage III non-small-cell lung cancer (NSCLC) which has not progressed following platinum-based chemoradiotherapy. A 63-year-old man presented to the emergency department with a 15-day history of increasing shortness of breath. Several months previously, he had been diagnosed with a poorly differentiated stage IIIB NSCLC. He had completed six cycles of chemotherapy with paclitaxel and carboplatin and four cycles of immunotherapy with durvalumab 13 days before his emergency hospital admission. Computed tomography (CT) imaging showed a large left-sided loculated hydropneumothorax suggestive of empyema, patchy opacification of the left lung, and a left upper lobe lung mass. Histology of the cell block from the pleural fluid and decorticated lung tissue showed hyphae suggestive of invasive *Aspergillus fumigatus*. Treatment with voriconazole resulted in clinical improvement. To our knowledge, this is the first reported case of pleural aspergillosis in a patient treated with durvalumab. However, the increasing use of immune checkpoint inhibitors in oncology requires increased awareness by clinicians of immune-related adverse events (irAEs) due to opportunistic infection.

## 1. Introduction

Durvalumab is a therapeutic monoclonal antibody that blocks the checkpoint inhibitor, programmed death ligand 1 (PD-L1), resulting in T-cell activation and an antitumor response [1, 2]. In 2017, durvalumab was approved for patients with unresectable stage III non-small-cell lung cancer (NSCLC) when the disease has not progressed following platinum-based chemoradiotherapy [3]. The approval for durvalumab followed the findings from the PACIFIC phase III clinical trial, which showed that adding durvalumab after chemoradiotherapy for stage III NSCLC resulted in significantly improved progression-free survival (PFS) [1].

In the past decade, the development of targeted therapy and immune therapy for NSCLC has progressed rapidly. Humanized therapeutic monoclonal antibodies that target growth factors, tumor-associated antigens, and immune checkpoint inhibitors are now increasingly used in clinical oncology. Fujita et al. recently reported that patients with NSCLC who received treatment with the anti-programmed cell death protein- (PD-) 1 antibody, nivolumab, were at an increased risk for occult infectious disease, especially patients with a history of diabetes mellitus [4]. The association between the use of immune therapy in oncology, including immune checkpoint blockade, and opportunistic infections has been termed immune-related adverse events (irAEs) [5].

There is a clinical spectrum of pulmonary aspergillosis that develops as a consequence of the interaction between the fungus and the host [6]. More common clinical presentations include invasive aspergillosis that develops in severely immunocompromised patients, chronic pulmonary aspergillosis that affects patients with chronic underlying lung disease, and allergic bronchopulmonary aspergillosis that affects patients with asthma and cystic fibrosis [6]. Pleural aspergillosis is a rare condition that has been reported to be due to *Aspergillus fumigatus*, *Aspergillus flavus*, and *Aspergillus terreus*, but coinfection of the pleural space with bacteria has also been reported [6]. Pleural aspergillosis has also been found in cases following pleural instrumentation and in the presence of a bronchopleural fistula [6]. The current European Organization for Research and Treatment of Cancer (EORTC-MSG) definitions recommend that positive cultures of *Aspergillus* spp. are obtained from lung specimens to confirm the diagnosis of infection [7].

A case is presented of pleural aspergillosis in a patient treated with durvalumab for stage IIIB non-small-cell lung cancer (NSCLC).

## 2. Case Report

A 63-year-old man who worked as a limousine driver presented to the emergency department of the Brooklyn Hospital Center, New York, with a 15-day history of increasing shortness of breath. Several months previously, he had been diagnosed with a poorly differentiated stage IIIB non-small-cell lung cancer (NSCLC) (squamous cell carcinoma). He had a previous episode of pleural effusion for which he received a thoracentesis. He had also received prednisone 50 mg for 5 days for a COPD exacerbation about a month before the hospital admission. He completed six cycles of chemotherapy with paclitaxel and carboplatin three months previously, before commencing four cycles of immunotherapy with durvalumab, an immune checkpoint inhibitor, which he completed 13 days before his emergency hospital admission.

He had a past medical history of chronic obstructive pulmonary disease (COPD), type 2 diabetes mellitus, hypertension, and chronic hepatitis C treated with sofosbuvir and ledipasvir (Harvoni). He had smoked one pack of cigarettes a week for 40 years and had formerly used alcohol but denied any illicit drug use. His father suffered from gout and hypertension, and his mother had a history of nephrectomy and had a permanent pacemaker.

Laboratory investigations on the day of admission showed reduced hemoglobin (Hb) 11.4 g/dl (normal range, 13.1–15.5 g/dl) and hematocrit 35% (normal range, 39–47%), with basic metabolic panel (BMP) tests within the normal range. An increased white blood cell (WBC) count 15.1 × 10^9^/l (normal range, 4.8–10.8 × 10^9^/l) included 87% neutrophils (normal range, 42.4–75.2%), 1.8% lymphocytes (normal range, 20.0–51.0%), and a platelet count of 323 × 10^9^/l (normal range, 130–400 × 10^9^/l). On the day following hospital admission, computed tomography (CT) of the chest showed a large left-sided loculated hydropneumothorax suggestive of empyema, patchy opacification in the left greater than the right lobe of the lung along with a left upper lobe lung mass. A pigtail catheter placed percutaneously under CT guidance drained an exudate containing 93% neutrophil polymorphs, which was sent for microbial culture. Intravenous antibiotic treatment commenced with ceftriaxone, which was then changed to vancomycin, piperacillin-tazobactam, and metronidazole, but with limited response. The patient had a persistent air leak and was noted to have a bronchopleural fistula, so on the tenth day after hospital admission, video-assisted thoracoscopic surgery (VATS) was performed.

**Figure 1:**
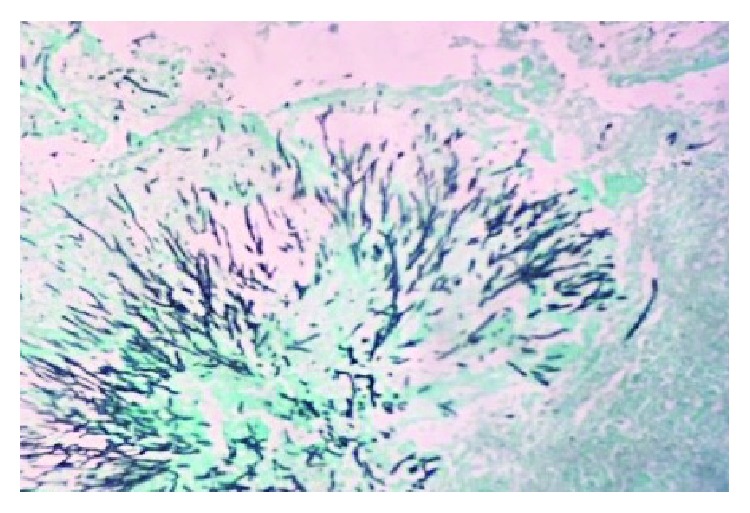
Photomicrograph of the pleural fluid cell block shows *Aspergillus fumigatus*, identified by histochemical staining. The acute angle of the branching hyphae (black) is distinctive (Gomori methenamine silver (GMS)). Magnification ×200.

Two weeks following hospital admission, fungal cultures from the pleural fluid, as well as from decorticated lung tissue, identified *Aspergillus fumigatus* (Figure 1). However, serology did not detect antibodies to *Aspergillus* spp. Treatment for aspergillosis commenced with voriconazole, a triazole antifungal agent. His clinical condition improved, and his WBC count stabilized at 11.1 × 10^9^/l. He was discharged home on linezolid and levofloxacin and a 60-day course of voriconazole.

## 3. Discussion

Durvalumab, pembrolizumab, and atezolizumab are therapeutic monoclonal antibodies to PD-1/PD-L1 checkpoint inhibitors currently licensed for the treatment of NSCLC [1–3]. This case has demonstrated that with the increasing use of immune checkpoint inhibitors in oncology clinicians should be aware of the association between opportunistic infections and immune therapy or immune-related adverse events (irAEs) due to opportunistic infection [4, 5].

Our patient contracted invasive aspergillosis while receiving durvalumab. However, the patient has COPD, diabetes mellitus, lymphopenia, and recent steroid exposures which are known risk factors for opportunistic infections including invasive aspergillosis (IA). In addition, the patient had a bronchopleural fistula, presumably related to prior thoracocentesis, that may have contributed to the IA in our patient.

Several recent studies have demonstrated the association between the use of immune checkpoint inhibitors and irAEs. The recent findings from a study that included a large cohort of patients with melanoma showed that approximately 7% of patients who received immune checkpoint inhibitors suffered from irAEs associated with severe atypical infection [8]. This study showed that opportunistic infections occurred most commonly in patients treated with infliximab and steroids [8]. The patient presented in this report had completed a course of steroids about a month before his recent hospital admission. The development of acute pulmonary tuberculosis in lung cancer patients following immune checkpoint inhibitor therapy without immunosuppressive treatment has been reported, which suggests the possible role of hypersensitivity, similar to immune reconstitution inflammatory syndrome (IRIS), leading to irAEs associated with opportunistic infection [9].

Fujita et al. retrospectively reviewed 167 patients with NSCLC treated with nivolumab and showed that the prevalence of lung infection was 19.2% (32 cases), of which 25 were bacterial, six were viral, and two cases were fungal [4]. This study showed that patients with NSCLC and a history of diabetes mellitus had a significantly increased prevalence of lung infection (OR, 3.61; 95% CI, 1.14–11.4; *p* = 0.028) [4]. These authors concluded that for patients with NSCLC receiving treatment with the checkpoint inhibitor, nivolumab, there was a significant risk for developing opportunistic infection and that diabetes mellitus was an independent risk factor [4]. The patient presented in this report had a history of diabetes mellitus. On the contrary, immune checkpoint inhibitors are being studied as adjunctive immunotherapy for immunosuppressed patients with IA, particularly AML patients [10].

According to the current European Organization for Research and Treatment of Cancer (EORTC-MSG) definitions, positive cultures of *Aspergillus* spp. from lung specimens, excluding BAL fluid, are recommended to make a proven diagnosis of fungal infection [7]. In this case, fungal organisms were isolated from the pleural fluid as well as from decorticated lung tissue, but no serum antibodies were detected. This finding raises the possibility that fungal organisms may have been a contaminant during the sampling procedure. Also, in immunosuppressed patients these antibodies may be negative. However, the findings from recent studies that have shown that both the *Aspergillus fumigatus*-specific IgM and IgG antibody assays and the galactomannan (GM) assay have limited value for the diagnosis of pulmonary aspergillosis [11]. The radiologic appearance in invasive aspergillosis is varied and nonspecific. It includes bronchial consolidation, centrilobular nodules, and bilateral bronchial dilation [12]. In our case, consolidation was seen which as above is a nonspecific finding. Also, in this case, the patient had a history of thoracocentesis and bronchopleural fistula, which may have been the source of infection.

These previous reports and the findings of this case highlight the importance of obtaining lung or pleural fluid samples for the identification of *Aspergillus fumigatus*, which can be identified directly by light microscopy using appropriate histochemical stains (Figure 1).

## 4. Conclusion

This report has presented a case of pleural aspergillosis in a patient treated with durvalumab, an immune checkpoint inhibitor, for stage IIIB non-small-cell lung cancer (NSCLC). To our knowledge, this is the first reported case of pleural aspergillosis in a patient treated with durvalumab. However, the increasing use of immune checkpoint inhibitors in oncology requires increased awareness by clinicians of immune-related adverse events (irAEs) due to opportunistic infection.

Longer follow-up and additional studies are required to determine causation between durvalumab treatment and invasive aspergillosis. Nonetheless, our case raises the awareness of opportunistic infections in patients treated with immune checkpoint inhibitors.
